# Comparison of health behaviors of adult women in Korea before and during the COVID-19 pandemic: secondary analysis of the Korea National Health and Nutrition Examination Survey 2019–2020

**DOI:** 10.4069/kjwhn.2022.08.22

**Published:** 2022-09-19

**Authors:** Mijong Kim, Hyunju Chae

**Affiliations:** 1 Department of Nursing, Hannam University, Daejeon, Korea; 2 Department of Nursing, Joongbu University, Geumsan, Korea

**Keywords:** COVID-19, Health behavior, Pandemics, Republic of Korea, Women

## Abstract

**Purpose:**

This study investigated the changes in the health-related behaviors of adult women in Korea during the coronavirus disease 2019 (COVID-19) pandemic.

**Methods:**

Data from the eighth Korea National Health and Nutrition Examination Survey (2019–2020) were analyzed. The participants were 4,848 women aged 19 to 64 years in 2019 and 2020. Data analysis using the complex sampling design was performed using SPSS 20.1.

**Results:**

Positive changes during the pandemic compared to before the pandemic in Korean adult women were found for improved subjective oral health perceptions (odds ratio [OR], 1.77; *p*<.001), increased moderate-intensity exercise in work and leisure activities (OR, 1.75; *p*<.001 and OR; 1.29, *p*=.004), and a decrease in secondhand smoke exposure at the workplace and in public places (OR, 0.64; *p*=.004 and OR, 0.60; *p*<.001). However, the following negative health behavior changes were found: decreased frequency of walking 5 days a week (OR, 0.81; *p*=.011) and an increase in unhealthy daytime sleep durations (OR, 1.40; *p*=006).

**Conclusion:**

Compared to before the COVID-19 pandemic, Korean adult women perceived their subjective dental health more positively during the COVID-19 pandemic, decreased their exposure to secondhand smoke at work and in public places, decreased walking, and increased sleep duration during the week. Since this study only compared data between 1 year before and after the start of the pandemic, it is necessary to investigate a longer period of time in the future. A future study should attempt to identify the factors related to changes in health behaviors caused by the pandemic.

## Introduction

The spread of coronavirus disease 2019 (COVID-19), caused by the severe acute respiratory syndrome coronavirus 2 (SARS-CoV-2) and considered the largest global health crisis of the 21st century, first started in Wuhan, China, in December 2019 and quickly spread around the world. On March 11, 2020, the World Health Organization (WHO) officially declared it a pandemic [1,2]. As of June 29, 2022, more than 545 million people have been infected worldwide and more than 6.33 million people have died of COVID-19 worldwide; thus, COVID-19 has been confirmed as a strongly contagious, fatal, and serious acute respiratory syndrome [2,3]. Although therapeutics and prophylactic vaccines for this novel virus have been developed and applied, gradually slowing the spread of the disease, the pandemic continues, causing 820,000 new infected cases per day worldwide as of the end of June 2022 [1,2].

Since the declaration of a pandemic, the WHO and governments of each country have responded with various policies and guidelines to prevent the spread of the virus. Although some treatments and vaccines have proven efficacy, SARS-CoV-2 is still a highly contagious and deadly virus that poses a threat to mankind. In addition to hand hygiene and mask wearing, social distancing and quarantine have become basic and essential practices in daily life [2,3]. In 2020, the first year of the COVID-19 pandemic, South Korea (hereafter, Korea) was gripped by fear and chaos. According to government policies such as remote learning, working from home, and bans on gatherings, except for essential livelihood and occupational activities, almost all daily life took place in the home, and work, school, and social activities were greatly restricted [3]. A series of unprecedented experiences, such as fear of COVID-19, financial difficulties due to job loss or lack of economic activity, psychological difficulties due to social isolation, and deprivation of indoor and outdoor sports activities except at home, caused heightened anxiety and stress. Overall, it is clear that COVID-19 affected the physical and mental health of the Korean people [1,4].

Meanwhile, as COVID-19 vaccination has been administered to 66.6% of the global population [5] and the acute transmission period has passed, considerable attention both in Korea and abroad is now being paid to its impact on various aspects of people who experienced the pandemic era. In addition to the impact of COVID-19 on physical and mental health [3,6], research is increasingly illuminating its impact on low-income and elderly population groups, who are considered vulnerable social groups in disasters [7,8].

This study investigated the health behaviors of adult women who could be vulnerable to health in an infection crisis called a pandemic. Traditionally and still today, women have been considered primary caregivers in the family, and they also play a major role in responding to family health issues and health care needs [9]. During the first year of the pandemic when almost all daily activities were conducted in the home due to the closure of childcare services and schools, it is thought that women took on a leading role as health managers in the home to protect their families from the spread of the COVID-19, as well as continuing to handle their responsibilities at work. In addition, the tasks of childcare and education, which had been shared by society, had to be carried out at home, placing women in a burdensome and difficult situation where they had to take care of and educate their children by themselves [7,10]. It is also probable that women mainly performed homeschooling and household chores such as meal preparation and cleaning for family members who worked from home. Under this aggravated burden, it is likely that women were not able to find time to exercise or were too fatigued, thereby affecting the health-related behaviors that they had previously maintained [10,11]. Compared to men, women have worked more in the face-to-face service industry, which is the first battleground for infectious diseases. Many women suffered economic difficulties due to the closure of the service industry as a whole, where women were mainly employed [11,12]. The economic difficulties could also have aggravated women’s difficulties in maintaining a healthy lifestyle.

Various studies have dealt with the effects of the pandemic on the economy, education, and culture in Korea and abroad [13-15]. However, studies on the relationship between the pandemic and health have generally focused on the pandemic’s effects on mental health [1,16], and the study participants were often limited to older adults or children and adolescents [8,17]. No study has yet investigated health behaviors in all Korean adult women during the pandemic through a comparison of data collected in the first year of the spread of COVID-19 with data obtained before the pandemic. As such, in this study, we attempted to understand changes in adult women’s health behaviors in association with the pandemic. This study compared and analyzed data from the 2019 Korea National Health and Nutrition Examination Survey (KNHANES), which was not affected by COVID-19, and from the 2020 KNHANES, which was conducted after the start of the COVID-19 pandemic, to examine changes in adult women’s health behaviors during the pandemic. Since the KNHANES contains data on representative samples of the Korean people, these surveys could provide reliable data on the health behaviors of Korean women in the first year of the pandemic.

The purpose of this study was to identify changes in the health behaviors of Korean women during the COVID-19 pandemic for use as basic data to establish strategies to improve women’s health in a similar global or national emergency in the future. The specific objectives of this study were as follows:

(1) To compare the general characteristics of Korean adult women before and after the start of the COVID-19 pandemic.

(2) To compare the health behaviors of Korean adult women before and after the start of the COVID-19 pandemic.

(3) To identify changes in health behaviors of Korean adult women during the COVID-19 pandemic compared to their health behaviors before the pandemic.

## Methods

Ethics statement: This study was a secondary analysis using the National Health and Nutritional Survey data and the data were received in an anonymous format. As such, Institutional Review Board approval was not sought, but the study adhered to the princi¬ples of the Helsinki Declaration.

### Study design

This study is a secondary analysis using 2 years of data from the eighth KNHANES conducted by the Korea Disease Control and Prevention Agency in 2019 (before the COVID-19 pandemic) and in 2020 (during the COVID-19 pandemic) to identify changes in the health behaviors of Korean adult women during the COVID-19 pandemic. The study was reported in compliance with the STROBE (STrengthening the Reporting of OBservational studies in Epidemiology) guidelines (https://www.strobe-statement.org/).

### Study participants

**Figure 1.**
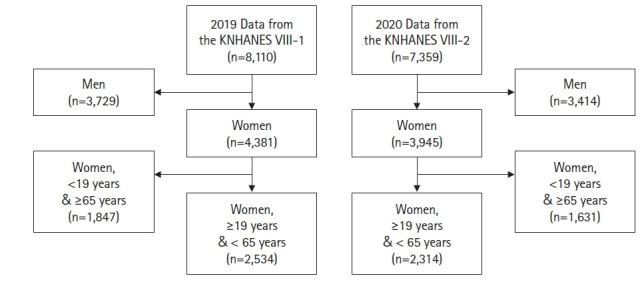
Flowchart of the study sample. KNHANES VIII, the eighth Korea National Health and Nutritional Examination Survey.

This study included a total of 4,848 adult women aged 19 to 64 years who participated in the first year of the eighth KNHANES (2019) conducted from January 2019 to December 2019 by the Korea Disease Control and Prevention Agency and the second year of the eighth KNHANES (2020) conducted from January 2020 to December 2020. In the first year of the eighth KNHANES (2019), there were a total of 8,110 participants, of whom 4,381 were women and 2,534 were adult women aged 19 to 64 years. In the second year of the eighth KNHANES (2020), the total number of participants was 7,359, of whom 3,945 were women and 2,314 were adult women aged 19 to 64 years (Figure 1).

### Measurements

#### General characteristics

The general characteristics analyzed in this study were age, total household income, education level classification, marital status, occupation classification, unemployment/no financial activity, the number of household members, and head of household status recorded in the health questionnaire of the KNHANES. *Age* was classified as 19–29 years, 30–39 years, 40–49 years, 50–59 years, and 60–64 years. *Income* was divided into five levels according to the monthly average equalized household income based on the household income investigated using an open-ended question: low, middle-low, middle, middle-high, and high. *Education* level was classified into ≤elementary school, middle school, high school, and ≥university.* Marital status* was classified depending on whether the participant had ever been married into unmarried and married, and the married category was further divided into “married, with spouse” for cases of spousal cohabitation and “married, without spouse” for cases of separation, bereavement, and divorce. Based on the *occupation* classification and unemployment/economic inactivity status, occupation was denoted as “no” for the unemployed and “yes” for the rest. *Household type* was classified as one-person for single-person households and multi-person for the rest. The *head of household* status was classified as “yes” or “no.”

#### Health behaviors

Health behaviors were assessed using the following data from the health questionnaire and health examination survey of the KNHANES: subjective health perception; self-perception of dental health status; perceived stress level; fulfillment of necessary medical services; history of unmet dental needs, health checkup, dental examination, cancer screening, and vaccination against influenza; frequency of drinking and binge drinking in one year; damage caused by someone else’s drinking in the past 1 year (noise, assault, sexual harassment, accidents at work or in daily life, drunk driving accidents, and others); history and current status of cigarette smoking; exposure to indoor secondhand smoke at home, work, or public places; history of obesity; high- and moderate-intensity physical activity (work, leisure); number of days of walking per week; number of days of strength training per week; time spent sitting per day; and average sleep duration per day on weekdays and weekends.

*Subjective perception of health:* Subjective perception of health and dental health were classified as “good” for responses of “good” and “excellent,” “ordinary” for a response of “ordinary,” and “poor” for responses of “poor” and “very poor,” referring to previous studies on perceptions of one’s own health and dental health [18,19]. Referring to previous studies on perceived daily stress [20], perceived stress was classified as “yes” for responses of “severe” and “a lot” of stress and as “no” for responses of “a little” and “hardly stressed.”

*Health care service use:* For experiences of missed medical care and dental treatment during the previous year, responses regarding whether hospital and clinic (excluding dentistry) visits were needed but skipped during the past year and experience in which dental treatment was necessary but not received were classified into “yes” or “no.” For health checkups and cancer screening, experiences of health checkups and cancer screening over the past 2 years were classified as either “yes” or “no,” and participants’ experiences of dental checkups were classified as “yes” or “no” based on the past 1-year history of dental checkups. Influenza vaccination was classified as “yes” and “no” according to whether or not participants had received an influenza vaccination during the last year.

*Alcohol consumption:* This was categorized using the frequency of drinking and the frequency of female binge drinking in the past year (the number of having more than five drinks on one occasion, regardless of whether it was *soju* or a Western alcoholic beverage) as “non-drinking” if there was no history of drinking in the past year, “drinking” if there was a history of drinking but no binge drinking in the past year, and “risky drinking” if there was a history of drinking and binge drinking in the past year. Damage caused by others’ drinking was classified as “yes” if there was at least one experience of damage caused by someone else’s drinking in the past year (noise, assault, sexual harassment, accidents at work or daily life, drunk driving accidents, and others) and “no” if there was no such experience.

*Smoking:* Smoking was classified as “yes” for daily and occasional smoking and “no” for past history of smoking but no current smoking and no lifetime history of smoking based on a question about participants’ cigarette smoking status. Exposure to secondhand smoke at home, work, and public places was classified as “yes” or “no” depending on whether or not they had inhaled other people’s cigarette smoke indoors at home, work, or a public place during the past 7 days.

*Body mass index:* Body mass index was classified as obese for stage 1, 2, and 3 obesity, overweight for one stage below obesity (≥23 and <25 kg/m^2^), “normal” for normal (≥18.5 and <23 kg/m^2^), and “low” for underweight (<18.5 kg/m^2^), according to standard classifications used to analyze the prevalence of obesity [21].

*Physical activity:* High-intensity physical activity (work) was classified as “yes” or “no” depending on whether participants engaged in high-intensity physical activities that cause intense shortness of breath and a fast heart rate for 10 minutes or more (e.g., lifting or carrying heavy objects, digging, working at a construction site, carrying objects upstairs, etc.). Moderate-intensity physical activity (work) was classified as “yes” or “no” depending on whether participants engaged in moderate-intensity physical activities that cause slight shortness of breath and a slightly fast heart rate for 10 minutes or more (carrying light objects, cleaning, and child-rearing [bathing, lifting, hugging, etc.]).

High-intensity physical activity (leisure) was classified as “yes” and “no” depending on whether participants engaged in high-intensity sports, exercises, and leisure activities (other than work-related activities) that cause intense shortness of breath and a fast heart rate for 10 minutes or more on a regular basis (running, rope jumping, hiking, basketball, swimming, badminton, etc.). Moderate-intensity physical activity (leisure) was classified as “yes” or “no” depending on whether participants engaged in moderate-intensity sports, exercises, and leisure activities that cause slight shortness of breath and a slightly fast heart rate for 10 minutes or more on a regular basis (running, weight training, golf, dance sports, Pilates, etc.).

*Walking and strength training:* For walking and strength training, data on the number of days of walking (walking for 10 minutes or over) and strength training (push-ups, sit-ups, dumbbells, weights, and iron bars, etc.) per week were used. Since the WHO recommends engaging in aerobic exercise at least five times a week and strength training at least two times a week for adults [22], the number of walking days per week was classified into ≤4 days and ≥5 days, and the number of days of strength training per week was classified into <2 days and ≥2 days.

*Sitting time:* This was analyzed using data on the time spent sitting on a daily basis (the time spent sitting or lying down per day, excluding the time for sleeping). Since the death rate is reported to increase when the sitting time is more than 10 hours per day [23], we classified open-ended responses for the usual amount of time spent sitting per day into <10 hours and ≥10 hours.

*Sleep duration:* Regarding weekday/weekend sleep duration, data on sleep duration on weekdays (or working days) and weekends (or nonworking days, the days before nonworking days) were used. Long or short sleep influences health. Referring to a previous study [24] that classified a sleep duration ≤6 hours as short sleep and a sleep duration ≥9 hours as long sleep, we classified open-ended responses for weekday and weekend sleep duration as ≤6 hours, 7–8 hours, and ≥9 hours.

### Data collection and analysis

The data of this study were basic data downloaded from the raw database of 2019 and 2020 after completing the statistical data user compliance requirement form and user information registration on the KNHANES website. The downloaded data were analyzed using IBM SPSS ver. 20.1 (IBM Corp., Armonk, NY, USA). Since the sample of the KNHANES was extracted using the two-step stratified cluster sampling, data analysis was performed using complex sample analysis considering the layer, cluster, and weight. Since the 2019 and 2020 data were combined, combined weights were calculated and applied. The Rao-Scott composite sample chi-square test was conducted to analyze differences to compare general characteristics and health behaviors of adult women in 2019 (before the COVID-19 pandemic) and in 2020 (during the COVID-19 pandemic). Complex sample logistic regression analysis was performed to examine the health behavior changes of adult women in 2020 (during the COVID-19 pandemic) and 2019 (before the COVID-19 pandemic).

## Results

### General characteristics

The largest number of participants belonged to the 50- to 59-year-old age group, accounting for 24.8% in 2019 and 24.7% in 2020, and the most common income group was “high,” in 29.0% of the participants in 2019 and in 29.2% in 2020. Almost half of the participants had university degrees or higher (48.8% in 2019 and 48.0% in 2020). Married and living with a spouse was the most common marital status, at 69.2% in 2019 and at 65.4% in 2020. The majority of participants were employed (61.3% in 2019 and 60.1% in 2020), and there were more multi-person households (92.8% in 2019 and 93.9% in 2020) than one-person households. Most participants were not the head of the household (67.7% in 2019 and 68.4% in 2020). None of the general characteristics of the participants showed statistically significant differences between 2019 and 2020 (Table 1).

### Differences in the health behaviors of adult women between before and during the pandemic

In adult women, health behaviors in 2020 (during the pandemic) showed differences from those in 2019 (before the pandemic) in terms of subjective perception of dental health, unmet dental needs, exposure to secondhand smoke at work and in public places, moderate-intensity work and leisure activities, walking, and weekday sleep duration.

Subjective dental health perceptions of “ordinary” and “poor” decreased from 2019 to 2020, whereas “good” perceptions increased from 13.8% to 22.0% (χ^2^=50.15, *p*≤.001). Missed dental treatment increased from 30.1% in 2019 to 42.6% in 2020 (χ^2^=65.97, *p*<.001). Exposure to secondhand smoke at work decreased from 12.1% in 2019 to 8.1% in 2020 (χ^2^=12.65, *p*=.004), and exposure to secondhand smoke in public places also decreased from 17.5% in 2019 to 11.2% in 2020 (χ^2^=38.63, *p*<.001). The percentage of women engaged in moderate-intensity work activities increased from 4.9% in 2019 to 8.2% in 2020 (χ^2^=21.26, *p*<.001). The percentage of women who engaged in moderate-intensity leisure activities increased from 23.5% in 2019 to 28.4% in 2020 (χ^2^=14.37, *p*=.004). Walking more than 5 days a week became less common, from 49.5% in 2019 to 44.2% in 2020 (χ^2^=12.87, *p*=.011). The percentage of women who slept 7 to 8 hours during weekdays decreased, while that of women who slept ≥ 9 hours increased from 9.1% in 2019 to 12.3% in 2020 (χ^2^=14.91, *p*=.007) (Table 2).

### Changes in the health behaviors of adult women during the pandemic

Complex sample logistic regression analysis was conducted for the health behaviors of adult women that showed significant differences between 2019 and 2020, including subjective perception of dental health, unmet dental needs, exposure to secondhand smoke at work and in public places, moderate-intensity work and leisure activities, walking, and sleep duration during the week. The complex sample logistic regression analysis was performed on individual variables by setting the year as the independent variable and the variables showing significant differences in the analysis presented above as the dependent variables to calculate the odds ratios. The health behaviors of adult women that significantly changed in 2020 (during the pandemic) compared to 2019 (before the pandemic) were subjective perception of dental health, exposure to secondhand smoke at work and in public places, moderate-intensity work and leisure activities, walking, and sleep hours during the week. However, no statistical significance was found for unmet dental needs.

The odds ratio of having good perceived dental health increased 1.77 times in 2020 (*p*<.001), and exposure to secondhand smoke at work decreased 0.64 times in 2020 (*p*=.004). Exposure to secondhand smoke in public places also decreased 0.60 times in 2020 (*p*<.001). Medium-intensity work increased 1.75 times in 2020 (*p*<.001) and medium-intensity leisure activities increased 1.29 times in 2020 (*p*=.004), but walking more than 5 days a week decreased by 0.81 times in 2020 (*p*=.011). The odds of a weekday sleep duration of ≥9 hours increased 1.40 times in 2020 (*p*=.006) (Table 3).

## Discussion

This is a secondary analysis using the data of the eighth KNHANES, conducted twice by the Korea Disease Control and Prevention Agency before and during the COVID-19 pandemic (2019–2020) to understand the changes in the health behaviors of Korean adult women during the COVID-19 pandemic.

In this study, there was an increase in the number of adult women perceiving their dental health as good in 2020 (during the pandemic) compared to 2019 (before the pandemic). In the second round of the sixth KNHANES, which was conducted in 2014, the subjective dental health perception of adult women over 19 years of age was good in 18.0% and ordinary or poor in 82.0% of participants [25]. Considering that 22.0% of the women perceived their dental health as good in the current study, it seems that there was an increasing tendency in adult women’s likelihood of perceiving their dental health as good during the pandemic compared to before the pandemic. In a study on dental health behaviors conducted among college students during the COVID-19 pandemic, the average frequency of brushing per day increased, as well as the use of oral care products such as dental floss and interdental toothbrushes, indicating a higher level of interest in dental hygiene [4]. Due to the lack of previous studies on the dental health behavior of adults during the pandemic, it is difficult to make a direct comparison. However, as mask wearing became mandatory as a simple and effective method to reduce the spread of COVID-19 [26], based on reports showing the main route of infection of COVID-19 was close contact (within 2 m) due to droplets that are emitted during coughing or speaking [27], this change in dental health behavior cannot be viewed as a phenomenon limited only to college students because dental hygiene management has become important due to mask wearing, as well as for COVID-19 prevention. Therefore, it can be inferred that adult women had more positive perceptions of their dental health status as they paid more attention to dental hygiene during the pandemic and took good care of it. Subjective dental health perception is related to objective dental health status [18,25] and the overall quality of life in adults [18]. Since COVID-19 has had a major influence on work, home, and social life throughout the world, it may have also affected dental health, but no studies have specifically investigated its effects on dental health behaviors [4]. Therefore, it is necessary to identify the changes in dental health behavior during the pandemic, investigate the reasons for these changes, and reflect them in future dental health management policies.

In this study, adult women showed a decrease in exposure to secondhand smoke at work and in public places in 2020 (during the pandemic) compared to 2019 (before the pandemic). This is consistent with a previous study [28] that reported that adolescents’ exposure to secondhand smoke in schools and public places decreased from 2019 to 2020 based on a comparison of the health behaviors of adolescents before and during the pandemic. However, in that previous study, exposure to secondhand smoke at home also decreased [28], showing a difference from the current study. In our study, exposure to secondhand smoke at home decreased from 43.9% in 2019 to 35.6% in 2020, but the difference was not statistically significant. Secondhand smoke, which refers to nonsmokers inhaling cigarette smoke exhaled by smokers [29], increases the risk of respiratory diseases, cardiovascular diseases, and cancer due to toxic substances [30]. It is estimated that 1% of annual mortality worldwide is related to secondhand smoke [31], and associations of secondhand smoke with mental illnesses such as depression, as well as physical diseases, have been reported [19]. Accordingly, many countries are making efforts to reduce the damage caused by secondhand smoke, and Korea is also expanding nonsmoking facilities to reduce the damage caused by secondhand smoke [27]. However, exposure to secondhand smoke continues even in nonsmoking facilities, such as exposure to ultrafine particles leaked from smoking rooms in nonsmoking facilities [32]. In this sense, the decrease in exposure to secondhand smoke after COVID-19 in the previous study [28] and the current study is significant. This decrease in exposure to secondhand smoke may be explained by a decrease in smoking during the COVID-19 pandemic, as reported in a previous study [33]. Furthermore, a decrease in face-to-face contact in public places due to mask wearing and social distancing may have also contributed to the reduced exposure to secondhand smoke. However, since this phenomenon occurred as a way to protect oneself from COVID-19, not a way to avoid the harm caused by secondhand smoke to others, continuing attention should be paid to exposure to secondhand smoke. In addition, as various measures associated with COVID-19 are lifted, it would be necessary to prepare for the possibility of an increase in exposure to secondhand smoke. In this study, exposure to secondhand smoke at home showed a tendency to decrease during the pandemic, but without statistical significance. Considering the increased frequency of working from home due to the pandemic, exposure to secondhand smoke at home can be an important threat to women’s health; therefore, it is necessary to clarify the actual situation and provide appropriate interventions. In particular, it may be necessary to focus on women who have no occupation as they spend more time at home and thus have higher chances of being exposed to secondhand smoke at home.

In this study, adult women showed increased moderate-intensity work and leisure activities in 2020 (during the pandemic) compared to 2019 (before the pandemic). Most preceding studies on changes in physical activity before and during the pandemic reported a decreasing tendency during the pandemic [34], unlike this study. This may have been due to differences in the measurement of physical activity in previous research and our study. In our study, moderate-intensity work was defined as an activity that caused at least 10 minutes of shortness of breath or a slightly rapid heartbeat, such as carrying light objects, cleaning, and activities performed as part of parenting. After the outbreak of COVID-19, as more office workers worked from home and more students took classes from home, they spent more time at home, and as a result, women also became directly responsible for family care and education [7,10]. This may explain the increase in moderate-intensity work, including cleaning and child-rearing activities, compared to pre-COVID-19 among women. In addition, in our study, moderate-intensity leisure activities, excluding work-related physical activities, were defined as sports, exercises, and leisure activities that cause continuous shortness of breath or slightly fast-paced heart rate for at least 10 minutes, such as light jogging, weight training, golf, sports dancing, and Pilates. Due to government policies to prevent COVID-19, the time spent at home has increased, while restrictions on work, school, and social activities have increased [3]. The frequency of at-home training has increased as the time spent at home has risen, especially among women [35]. Considering that weight training was included in moderate-intensity leisure activities in this study, the increase in at-home training may have resulted in the increased moderate-intensity leisure activities.

In contrast, the decrease in walking during the pandemic in this study is consistent with a previous study [34] that also reported a decrease in physical activity. The decrease in physical activity during the pandemic may have resulted from social distancing measures implemented by the government to stop the spread of COVID-19 in community [4,34]. Social distancing is a basic method to curb the spread of COVID-19 [34], but it has resulted in negative phenomena such as decreased physical activity and increased sedentary behavior [36]. Research related to physical activity has emerged as an urgent public health topic during the COVID-19 pandemic [37], as decreased physical activity has been reported to be related to various factors such as sex, age, occupation, stress, and obesity [38]. In the COVID-19 pandemic, to maintain the same level of physical activity as before the pandemic, physical activity at home is encouraged [20]. Studies have described using web-based data for physical activity at home [39] and have found that at-home training increased during the pandemic among adolescents [40]. Therefore, it will be necessary to develop and disseminate physical activity programs that can be used at home in consideration of individual characteristics. In addition, with the recent easing of measures associated with COVID-19, including social distancing, it is necessary to identify resultant changes in physical activity and encourage people to appropriately combine physical activities that can be done at home with those that can be done outside of the home depending on the situation.

In this study, the percentage of adult women who had 9 hours or more of sleep per day increased in 2020 (during the pandemic) compared to 2019 (before the pandemic). A previous study reported that the COVID-19 pandemic increased sleep duration [41], consistent with this study. This can be attributed to the increased time spent at home due to social distancing and the increased frequency of working from home due to COVID-19 [42]. However, in adolescents, the sleep satisfaction rate [28] and recovery from sleep fatigue [43] increased during the pandemic, improving the quality of sleep, whereas the quality of sleep decreased in adults during the pandemic [41,42]. This can be attributed to the increase in stress for adults due to the economic blows caused by COVID-19 [43] or increased child-rearing hours due to school closures [42]. Therefore, although only sleep duration was investigated in the current study, the quality of sleep should also be measured and the causes of changes in sleep patterns should be identified in further studies to understand the influence of the pandemic on sleep and provide appropriate interventions.

Taken together, this study found that there were both positive and negative changes during the COVID-19 pandemic in adult women in Korea. Specifically, they perceived their subjective dental health more positively during the COVID-19 pandemic than before the COVID-19 pandemic, their exposure to secondhand smoke at work and in public places decreased, and their moderate-intensity work and leisure activities increased, while walking decreased, and sleep duration during the week increased. Therefore, it is necessary to provide guidelines for physical activities such as walking according to the severity of the pandemic so that appropriate physical activities can be performed even during the pandemic. It is also advised to encourage the maintenance of appropriate sleep duration through education on appropriate sleep duration and lack of sleep or oversleeping. In addition, in order to ensure that the positive changes caused by the pandemic, such as reduced exposure to secondhand smoke, are maintained, it is necessary to strengthen education on secondhand smoke and identify factors related to reduction in exposure to secondhand smoke to reflect in future health management policies.

This study has the following limitations. In this study, data from 2019 (before the COVID-19 pandemic) and 2020 (during the COVID-19 pandemic) were compared. Since only data from 1 year before and during the pandemic were compared, it is difficult to conclude that the observed changes were due to the COVID-19 pandemic. We suggest that a study comparing 2 years of data from before and during the COVID-19 pandemic should be conducted. In addition, this study only compared the observed differences before and during the COVID-19 pandemic without identifying the factors that caused these differences. Therefore, future studies should investigate factors related to the occurrence of these differences due to the COVID-19 pandemic. Lastly, this study did not take into account differences according to women’s life cycle, since all women aged 19 to 64 years were the study participants. Therefore, future research should explore differences by age group.

## Figures and Tables

**Table 1.** General characteristics

Characteristic	Categories	2019 (n=2,534)		2020 (n=2,314)		χ^2^ (*p*)
		n^†^	%^‡^ (SE)	n^†^	%^‡^ (SE)	
Age (years)	19–29	362	20.5 (1.1)	401	20.9 (1.1)	0.58 (.980)
	30–39	503	20.4 (1.2)	430	19.8 (1.1)	
	40–49	630	23.6 (1.1)	550	23.4 (1.2)	
	50–59	697	24.8 (0.9)	580	24.7 (1.0)	
	60–64	342	10.7 (0.7)	353	11.2 (0.7)	
Income	Low	161	6.2 (0.7)	153	6.2 (0.7)	6.58 (.560)
	Middle-low	415	15.7 (1.0)	321	13.2 (1.0)	
	Middle	563	22.5 (1.2)	539	23.3 (1.1)	
	Middle-high	661	26.7 (1.1)	642	28.1 (1.2)	
	High	721	29.0 (1.7)	655	29.2 (1.9)	
Education	≤Elementary school	179	6.2 (0.6)	141	4.8 (0.6)	6.13 (.366)
	Middle school	186	6.3 (0.6)	177	6.3 (0.6)	
	High school	926	38.7 (1.3)	867	40.9 (1.5)	
	≥University	1,149	48.8 (1.6)	1,022	48.0 (1.7)	
Marriage	Unmarried	452	22.4 (1.2)	532	26.5 (1.2)	11.03 (.054)
	Married, without spouse	253	8.4 (0.7)	239	8.1 (0.7)	
	Married, with spouse	1,829	69.2 (1.3)	1541	65.4 (1.4)	
Occupation	No	979	38.7 (1.3)	899	39.9 (1.4)	0.78 (.522)
	Yes	1,555	61.3 (1.3)	1,415	60.1 (1.4)	
Household type	One-person	207	7.2 (0.7)	169	6.1 (0.8)	2.05 (.366)
	Multi-person	2,327	92.8 (0.7)	2,145	93.9 (0.8)	
Household head	No	1,672	67.7 (1.2)	1,532	68.4 (1.3)	0.25 (.709)
	Yes	862	32.3 (1.2)	782	31.6 (1.3)	

† Unweighted and valid frequency,

‡ valid percentage.

**Table 2.** Differences in health behaviors in 2019 and 2020

Characteristic	Categories	2019 (n=2,534)		2020 (n=2,314)		χ^2^ (*p*)
		n^†^	%^‡^ (SE)	n^†^	%^‡^ (SE)	
Perceived health status	Poor	387	15.4 (0.9)	389	17.7 (1.0)	4.89 (.191)
	Ordinary	1,328	54.2 (1.2)	1,183	53.1 (1.4)	
	Good	728	30.4 (1.1)	637	29.2 (1.1)	
Perceived dental health status	Poor	765	34.4 (1.4)	662	30.5 (1.5)	50.15 (<.001)
	Ordinary	1,154	51.8 (1.7)	961	47.5 (2.0)	
	Good	300	13.8 (1.2)	472	22.0 (1.7)	
Perceived stress	No	1,748	68.4 (1.0)	1,546	66.8 (1.2)	1.39 (.303)
	Yes	757	31.6 (1.0)	758	33.2 (1.2)	
Unmet medical needs	No	2,229	92.9 (0.6)	1,948	91.7 (0.8)	2.59 (.183)
	Yes	187	7.1 (0.6)	184	8.3 (0.8)	
Unmet dental needs	No	1,376	69.9 (1.4)	1,094	57.4 (1.4)	65.97 (<.001)
	Yes	625	30.1 (1.4)	816	42.6 (1.4)	
Medical checkup	No	779	34.8 (1.2)	679	32.6 (1.1)	2.57 (.182)
	Yes	1,662	65.2 (1.2)	1,527	67.4 (1.1)	
Dental checkup	No	1,402	55.4 (1.2)	1,293	55.3 (1.2)	0.01 (.936)
	Yes	1,103	44.6 (1.2)	1,011	44.7 (1.2)	
Cancer screening	No	801	36.8 (1.2)	801	38.3 (1.2)	1.15 (.377)
	Yes	1,640	63.2 (1.2)	1,406	61.7 (1.2)	
Influenza vaccination	No	1,516	62.9 (1.3)	1,318	60.6 (1.5)	2.52 (.257)
	Yes	925	37.1 (1.3)	899	39.4 (1.5)	
Drinking	Non-drinking	676	26.3 (1.0)	655	26.3 (1.2)	0.44 (.862)
	Drinking	795	30.1 (1.1)	673	29.3 (1.2)	
	Risky drinking	1,033	43.6 (1.2)	976	44.4 (1.2)	
Damage caused by others’ drinking	No	2,304	92.1 (0.6)	2,109	92.0 (0.6)	0.01 (.965)
	Yes	201	7.9 (0.6)	195	8.0 (0.6)	
Smoking	No	2,351	93.5 (0.7)	2,164	93.7 (0.6)	0.126 (.798)
	Yes	154	6.5 (0.7)	140	6.3 (0.6)	
Secondhand smoke exposure	No	201	56.1 (3.7)	222	64.4 (3.0)	5.26 (.089)
in the home	Yes	172	43.9 (3.7)	134	35.6 (3.0)	
Secondhand smoke exposure	No	1,337	87.9 (1.0)	1,273	91.9 (0.9)	12.65 (.004)
in the workplace	Yes	177	12.1 (1.0)	110	8.1 (0.9)	
Secondhand smoke exposure	No	2,072	82.5 (1.2)	2,050	88.8 (0.9)	38.63 (<.001)
in public places	Yes	433	17.5 (1.2)	254	11.2 (0.9)	
Body mass index	Low weight	156	7.2 (0.7)	143	6.7 (0.7)	8.08 (.189)
	Normal	1,224	50.3 (1.3)	1,048	46.7 (1.4)	
	Overweight	467	17.4 (0.8)	431	18.6 (1.4)	
	Obese	649	25.1 (1.1)	666	27.9 (1.3)	
Physical activity:	No	2,422	99.4 (0.2)	2,188	99.2 (0.2)	0.64 (.508)
high-intensity work	Yes	18	0.6 (0.2)	19	0.8 (0.2)	
Physical activity:	No	2,313	95.1 (0.5)	2,043	91.8 (0.8)	21.26 (<.001)
moderate-intensity work	Yes	127	4.9 (0.5)	164	8.2 (0.8)	
Physical activity:	No	2,252	92.0 (0.7)	2,013	90.6 (0.8)	3.17 (.147)
high-intensity leisure	Yes	188	8.0 (0.7)	194	9.4 (0.8)	
Physical activity:	No	1,858	76.5 (1.1)	1,611	71.6 (1.2)	14.37 (.004)
moderate-intensity leisure	Yes	582	23.5 (1.1)	596	28.4 (1.2)	
Walking (days/week)	≤4	1,282	50.5 (1.3)	1,214	55.8 (1.5)	12.87 (.011)
	≥5	1,159	49.5 (1.3)	993	44.2 (1.5)	
Weight training (days/week)	<2	2,068	84.6 (0.8)	1,840	83.0 (0.9)	2.32 (.173)
	≥2	373	15.4 (0.8)	367	17.0 (0.9)	
Sitting time (hours/day)	<10	1,453	55.8 (1.4)	1,242	52.6 (1.3)	5.10 (.118)
	≥10	1,081	44.2 (1.4)	1,072	47.4 (1.3)	
Weekday sleep duration (hours/day)	≤6	1,026	40.3 (1.2)	949	40.7 (1.1)	14.91 (.007)
	7–8	1,283	50.6 (1.3)	1,104	47.0 (1.1)	
	≥9	225	9.1 (0.8)	261	12.3 (0.9)	
Weekend sleep duration (hours/day)	≤6	633	24.5 (1.0)	603	24.7 (0.9)	4.55 (.171)
	7–8	1,280	49.4 (1.3)	1,095	46.9 (1.2)	
	≥9	621	26.1 (1.1)	616	28.6 (1.0)	

† Unweighted and valid frequency,

‡ valid percentage.

**Table 3.** Changes in health behaviors in 2020 compared to 2019

Variable	Categories	Odds ratio	95% CI	*p*
Perceived dental health status^†^	Good	1.77	1.35–2.31	<.001
Unmet dental needs^†^	Yes	1.20	0.92–1.56	.183
Secondhand smoke exposure in the workplace^†^	Yes	0.64	0.47–0.87	.004
Secondhand smoke exposure in public places^†^	Yes	0.60	0.47–0.76	<.001
Physical activity: moderate-intensity work^†^	Yes	1.75	1.28–2.38	<.001
Physical activity: moderate-intensity leisure^†^	Yes	1.29	1.08–1.53	.004
Walking^†^	≥5 days/week	0.81	0.69–0.95	.011
Weekday sleep duration^†^	≥9 hours/day	1.40	1.10–1.78	.006

† Dummy variable references were perceived dental health (poor and ordinary), unmet dental needs (no), secondhand smoke exposure in the workplace (no), secondhand smoke exposure in public health (no), physical activity: moderate-intensity work (no), physical activity: moderate-intensity leisure (no), walking (<5 days/week), weekday sleep duration (<9 hours/day).
